# Finite Element Analysis of Insertion Angle of Absorbable Screws for the Fixation of Radial Head Fractures

**DOI:** 10.1111/os.12797

**Published:** 2020-09-30

**Authors:** Guang‐ming Xu, Zi‐yang Liang, Wei Li, Zheng‐zhong Yang, Zhi‐bin Chen, Jie Zhang

**Affiliations:** ^1^ Department of Orthopaedics Shenzhen Pingle Orthopedic Hospital & Shenzhen Pingshan Traditional Chinese Medicine Hospital Shenzhen China; ^2^ First Clinical Medical College Guangzhou University of Chinese Medicine Guangzhou China

**Keywords:** Absorbable implant, Finite element analysis, Radius fracture

## Abstract

**Objective:**

To investigate the biomechanical effects of different insertion angles of absorbable screws for the fixation of radial head fractures.

**Methods:**

The finite element models used to simulate the fractures were created based on CT scans. Two absorbable screws were used to fix and maintain the stability of the fracture, and the angles between the screws were set to 0°, 15°, 30°, 45°, 60°, 75°, and 90°. A downward force of 100 N was applied at the stress point, which was coupled with the surface, and the distal radius was limited to six degrees of freedom. The direction and location of the applied force were the same in each model. The values of the von Mises stress and peak displacements were calculated.

**Results:**

Under the applied load and different screw angles, the maximum von Mises stress in the screws was concentrated on the surface contacting the fracture surfaces. The maximum von Mises equivalent stress in the screw decreased when the angle increased from 0° (19.54 MPa) to 45° (13.11 MPa) and increased when the angle further increased to 90° (24.63 MPa). The peak displacement decreased as the angle increased from 0° (0.19 mm) to 45° (0.15 mm) and increased when the angle further increased to 90° (0.25 mm).

**Conclusion:**

The computational stress distribution showed that fixation with absorbable screws is safe for patients. Moreover, the minimum von Mises stress and displacements were generated when the angle between the screws was 45°; hence, this setting should be recommended for Mason type II radial fractures.

## Graphical abstract

This current study uses a finite element model to study the influence of absorbable screw designs in type II radial fractures. The 45° angle between two screws provided the optimal biomechanical effect, and generated wider stress distribution, and the minimum stress and displacement in screw fixation.

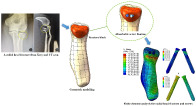

## Introduction

Radial head fractures account for 3% to 5% of fractures in the elbow joint, which are infrequent in adults^1^, ^2^, ^3^, ^4^. This type of fracture is commonly caused by an indirect external force transmitted through the radial axis of the wrist to the elbow joint, and the elbow is subjected to excessive eversion stress due to the brace angle, which causes an impact between the humeral head and the radial head that generates the fracture. This type of fracture can also be caused by direct violence. The radial head plays an important role in carrying axial loads and stabilizing the elbow joint^5^, ^6^. The Mason classification system is used to classify radial head fractures. For common type II radial head fractures (which have greater than 2 mm of displacement and involve at least 30% of the radial head), pins or Herbert screws have been used in the past for open reduction and internal fixation (ORIF)^7^. Murat Demiroglu *et al*.^8^ evaluated the effectiveness of treating Mason II radial head fractures through fixation with a Micro Acutrak 2 Screw and found that anatomical reduction of type II radial head fractures through open surgery and fixation with screws can have favorable results.

The mechanical characteristics of absorbable poly‐L‐lactide acid (PLLA) are very similar to those of cortical bone^8^, ^9^. Orthopaedic implant materials have been widely used in many types of orthopaedic surgeries, such as for radial fractures, calcaneal fractures, and proximal humeral fractures. Using orthopaedic implant materials can avoid secondary operations, reducing patients’ psychological stress and surgical trauma response; moreover, due to the characteristics of absorbable materials, the fixation of a fracture can be better assessed in X‐rays. Tarallo *et al*.^10^ introduced a technique that uses absorbable pin fixation to provide adequate strength and rigidity for the treatment of Mason type II and Mason type III radial head fractures; they compared the results achieved with absorbable pin fixation with those achieved with mini‐screw fixation. However, a higher rate of secondary displacement of the fracture fragments was reported among subjects who had been treated using absorbable pins. Previous studies did not use a finite element (FE) model to assess the biomechanical stability of radial fractures fixed with absorbable screws, which is necessary to determine the biomechanics of the application.

In the last decade, the FE method (FEM) has increasingly been used in the field of orthopaedic research. When using the FEM, the model of a complex structure can be divided into a finite number of elements. Then, after defining the geometric shape, material properties, and boundary conditions in the model and imposing the selected loading conditions, the responses in each element can be assessed. This approach can replicate the biomechanics observed in traditional experiments and can help determine locations where failure may occur. Moreover, FE analysis (FEA) can accurately calculate the displacement distribution and stress–strain response of each element in the model. FEA has been widely used in many research fields, such as studies on the humerus, distal radius, knee joint, and ligaments^11^, ^12^, ^13^, ^14^. For example, Sergey Strafun *et al*.^13^ constructed an FE model to show that the radial head is the primary stabilizing structure of the elbow joint and that the medial collateral ligament is the second structure responsible for valgus stability of the elbow joint. Using FEA, Tan *et al*.^14^ found that longitudinal displacement of the radial head causes it to slip out of the annular ligament while the ligament remains intact in Monteggia fractures. However, there has been no FEA report concerning the primary stability of radial head fractures fixed with absorbable screws. The purpose of the present study is to develop an FE model of a human elbow joint and to investigate the effects of different angles of absorbable screw fixation for radial head fractures.

## Materials and Methods

### *Participant and* *CT* *Scanning*

**Fig. 1:**
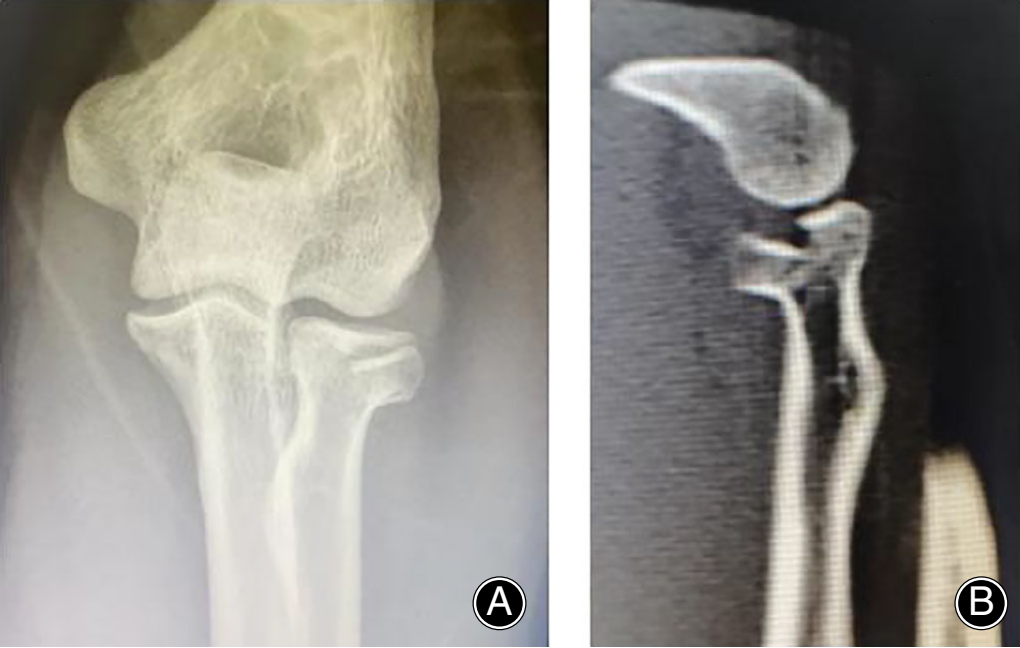
The common characteristics of the Mason type II radial head fractures were obtained by X‐ray and CT images. The CT machine was used to scan the elbow. A total of 380 slices (0.5 mm thickness) tomograms were saved in the dicom format.

The present study was approved by the ethics committee of our institution and written informed consent was obtained from the participants. To investigate the effects of different angles of absorbable screw fixation for radial head fractures, FE models of the radial head were created based on CT results. The common characteristics of the Mason type II radial head fractures according to X‐ray and CT images were more than 2 mm of displacement and involvement of at least 30% of the radial head (Fig. 1). For this study, the left elbow of a healthy Chinese man (age: 34 years; body weight: 73 kg; height: 170 cm) was CT scanned. He had no previous history of elbow joint injury, and X‐ray examination of the elbow joint had preliminarily excluded radial head trauma, arthritis, bone tumor, and other diseases. CT scans were undertaken in the ShenZhen PingLe Orthopedic Hospital (Guangdong, China) on 21 January 2020 using a 64‐slice CT scanner (Siemens Medical Systems). With the consent of the participants, a total of 380 slices (0.5‐mm thickness) of DICOM images were obtained.

### *Three‐Dimensional Reconstruction of Radius Head*

First, the CT images were imported into the image processing software MIMICS 19.0 (Materialize, Leuven, Belgium), where the primary radial head geometry was analyzed. Second, the geometric model was imported into Geomagic Studio (Geomagic, USA) to complete the smooth and curved structure and to construct a solid model of the radial head. Third, a Mason type II radial head fracture was modeled in SolidWorks (SolidWorks, Dassault Systèmes, USA). Two absorbable screws with different angles, which were used for fixing the radial head fractures, were developed according to the clinical operation requirements. The screws had a diameter of 2 mm and a length of 18 mm.

### *Boundary and Loading Conditions*

**Fig. 2:**
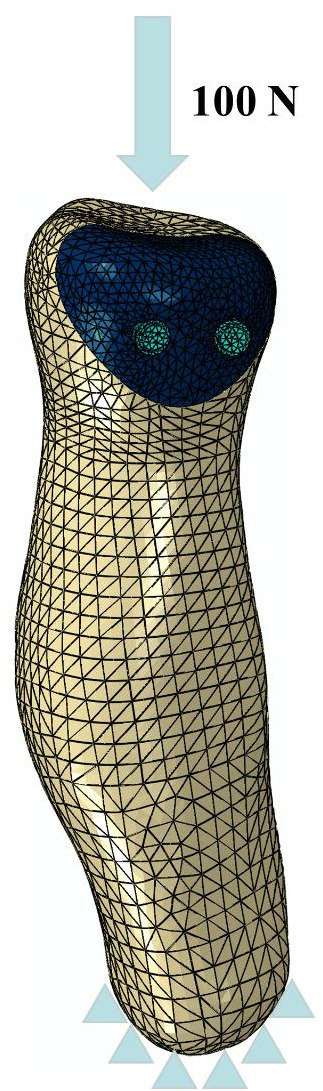
In the segmentation module, the bone structure was selected by threshold selection to draw erase, to calculate three dimensions, to smooth surfaces to perform other editing processes, and to obtain the corresponding position of the mark. Then, the three‐dimensional model was reconstructed and the vertical downward force of 100 N was applied at the stress point. It was coupled with the surface, while the distal radius was limited to six degrees of freedom.

The model was imported into the FEA software Abaqus 13.0 (Dassault Systèmes, USA). In the model, the interaction between the bone surfaces was simulated with “surface‐to‐surface contact,” and the coefficient of friction was set to μ = 0.1. The contact between the radial head and the screw was defined as bonded. Two absorbable screws were used to fix the fracture and maintain the stability of the fracture, and the included angles of the two screws were set to 0°, 15°, 30°, 45°, 60°, 75°, and 90°. The values of these loads and load locations were selected in accordance with previously published studies. A vertical downward force of 100 N was applied at the stress point^15^, ^16^. It was coupled with the surface, while the distal radius was limited to six degrees of freedom. The loading and boundary conditions are detailed in Fig. 2.

### *Preprocessing of Finite Element Modeling*

In accordance with previously published reports, to ensure accurate measurements, the cancellous and cortical bones and the implants were defined as homogenous, isotropic, and linearly elastic in Abaqus, and then an FE model with nonuniform material properties was successfully built. The mechanical properties of the bones and absorbable screws were selected from previously published reports^13^, ^17^ and are listed in Table 1. The thickness of the cortical bone was set to 0.5 mm. Based on the relevant literature, a material attribute assignment was carried out to construct a three‐dimensional FE model of the radial head. Sergey Strafun *et al*.^13^ previously conducted a mesh convergence test of a human elbow joint model and found that mesh refinement had little effect on the results when increased to a certain extent. In this study, we found that the difference in value was small when the mesh consisted of 86,000 elements (linear tetrahedrons).

**TABLE 1:** Mechanical properties of cortical and cancellous bones and absorbable screws

Material name	Young's modulus (MPa)	Poisson's ratio
Cortical bone	18,000	0.3
Cancellous bone	400	0.26
Absorbable implants	1400	0.2

### *Observational Factors*

The direction and location of the applied force were the same in each model. The values of the von Mises stress and peak displacements were calculated using the FEA software Abaqus 13.0 (Dassault Systèmes, USA).

## Results

### *Von Mises Stress Distribution*

**Fig. 3:**
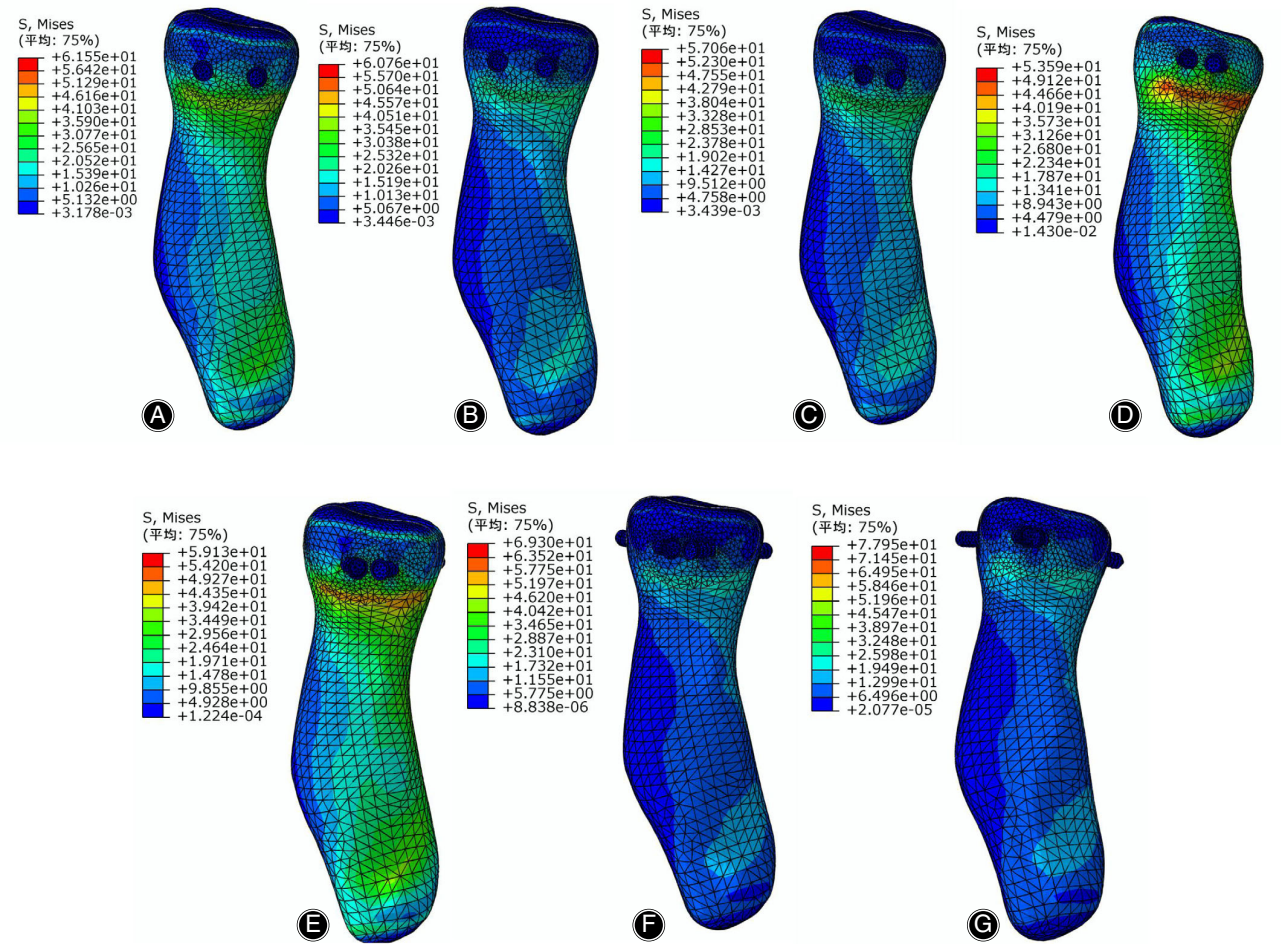
Stress distribution of the radial head under different insertion angles of absorbable screws: (A) The angle set to 0°; (B) the angle set to 15°; (C) the angle set to 30°; (D) the angle set to 45°; (E) the angle set to 60°; (F) the angle set to 75°; and (G) the angle set to 90°.

**Fig. 4:**
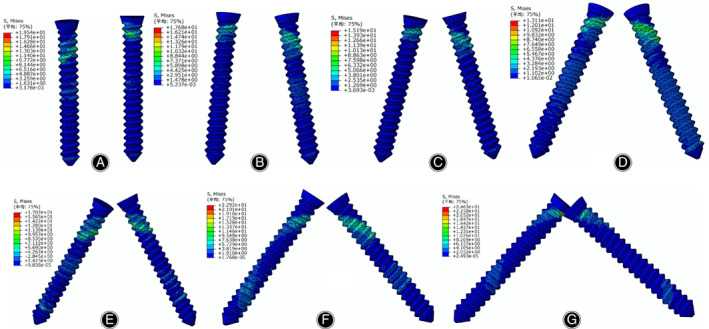
Stress distribution of the screws under different insertion angles: (A) The angle set to 0°; (B) the angle set to 15°; (C) the angle set to 30°; (D) the angle set to 45°; (E) the angle set to 60°; (F) the angle set to 75°; (G) the angle set to 90°.

The von Mises stress distributions in the radial head and screws are listed in Figs 3 and 4, respectively. According to the stress distribution in the radial head model in Fig. 3, the maximum equivalent stress in the bone tissue was concentrated on the lower boundary surface of the fracture block. At different angles between the two screws in Fig. 4, the stress in the screws was concentrated on the surface in contact with the fracture surfaces. The maximum von Mises equivalent stress in the screw decreases when the angle changes from 0° (19.54 MPa) to 45° (13.11 MPa). In contrast, when the angle changes from 45° to 90°, the maximum von Mises equivalent stress of the screw increases, reaching a maximum value of 24.63 MPa.

### *Peak Displacement Distribution*

**Fig. 5:**
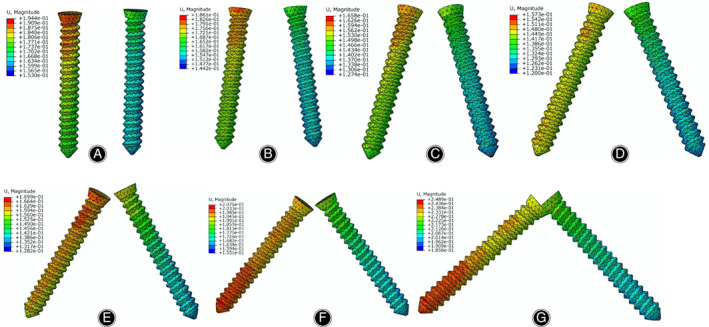
Peak displacements of screws under different insertion angles. (A) The angle set to 0°; (B) the angle set to 15°; (C) the angle set to 30°; (D) the angle set to 45°; (E) the angle set to 60°; (F) the angle set to 75°; and (G) the angle set to 90°.

The peak displacements of the screws are shown in Fig. 5. This results show that the peak displacement of the screw decreases when the angle changes from 0° (0.19 mm) to 45° (0.15 mm). In contrast, when the angle changes from 45° to 90°, the peak displacement of the screws increases, reaching a maximum value of 0.25 mm.

## Discussion

### *Treatment of Radial Head Fractures*

The physiological role of the radial head is to transfer stress and keep the outer part of the elbow joint in a stable state, which plays an important role in maintaining the stability and functionality of the elbow joint. The treatment of radial head fractures has evolved from nonsurgical treatment at the beginning to internal fixation, removal of the radial head, and replacement with artificial prostheses. However, most treatment options are based on clinical practice. Hence, it is critical to determine the degree of injury for evaluating treatment effectiveness, as the actual symptoms are often more severe than those seen during imaging. Treatment of radial head fractures should aim to restore the flexion and extension of the elbow and rotation of the forearm, stabilize the elbow joint, and reduce the incidence of post‐traumatic arthritis. If Mason II radial head fractures (which have greater than 2 mm of displacement and involve at least 30% of the radial head) are treated conservatively, joint stiffness is the most common complication limiting forearm rotation, making supination difficult to restore. The deformation of the radial head after fracture or the effect on the medial half of the articular surface can result in limited forearm rotation, resulting in pain during movement and even traumatic arthritis of the brachioradial and superior radioulnar joints. Mason II type radial head fractures require hand surgery therapy, surgical internal fixation operation diversity, and different architectural effects. For this type of intra‐articular radial head fracture, internal fixation can involve the humeroulnar joint and radioulnar joints, and most of the internal fixation should be in a “safe haven.” For internal fixation of radial head fractures, attention must be paid to the concept of the “safe zone.” This zone is approximately 45° posterior to and 65° anterior to the radial head, which serves as the center of the forearm. Caputo *et al*.^18^ defined the “safe zone” of the radial head as follows: the peripheral edge of the radial head was approximately 113° (106°–120°) and did not involve the upper radioulnar joint. Surgical treatment is generally recommended for Mason type II radial head fractures, and the surgical method and internal fixation are usually determined according to intraoperative findings and individual characteristics^19^, ^20^. Lipman *et al*.^19^ used a tripod technique with headless compression screws to fix radial head fractures. According to a systematic review and meta‐analysis of clinical outcomes, Zwingmann^20^ showed that the best treatment option for type II fractures was ORIF using screws or biodegradable (polylactide) pins, with an overall success rate of 98%.

### *Absorbable Implant for Radial Head Fractures*

Absorbable implants have been widely used in orthopaedic surgery^21^, ^22^, ^23^. This approach is effective and safe for fixation of radial head fractures because the modulus of absorbable screws is similar to that of cortical bone. Absorbable screws are composed of a medical‐grade polymer (a new internal fixation material) that is mainly used for cancellous bone and intra‐articular fractures. This material has obvious advantages, including good biological compatibility, no obvious rejection or infection in the body, and biodegradability in water and carbon dioxide; moreover, this material is eventually fully absorbed by the human body. In addition, secondary surgery (especially for elbow joints) caused by complications, such as infection and tissue adhesion, can be avoided, greatly reducing the pain and medical costs for patients. Because the absorbable rod does not have an expanded end, there is no need for a reaming head. The absorbable rod can undergo transverse expansion and longitudinal contraction and produce an automatic compression effect, which makes the fixation more firm; hence, these devices can be used for internal fixation of radial head fractures. Pelto *et al*.^23^ showed that using polylactide pins for fixation of radial fractures was reliable based on postoperative functional and radiographic results and was feasible for the treatment of radial head fractures. There have been some biomechanical studies on the fixation of radial fractures^24^, ^25^. Wagner *et al*.^24^ compared the stabilization of biodegradable pins and titanium screws using the fixation method for radial fractures. Shi *et al*.^25^ found that the divergent screw orientation was the most stable and had the greatest control for Mason type II fractures. These studies used cadaveric or physical models to simulate fractures and fixation. However, these approaches also have limitations, with some parameters that cannot be measured. In these test studies, because one implant hole cannot be repeated for other screws or different test conditions, many screw holes must be made in the same bone or repeated with other bones. This is especially problematic for human radial heads, as the bone density is not uniform even in the same radial tissue. Therefore, the difference in the screw angle may affect the accuracy of the mechanical test results. This study performed an FEA, and the stress distribution in the complex material of the radial head was simulated and evaluated using computer software, which has obvious advantages and can be fixed at precise angles to provide more standardized and repeatable data. Moreover, this study used the elbow joint CT data from patients with radial head injuries to establish a three‐dimensional anatomical model of the radial head, which could accurately reflect the anatomical shape and bone condition of the radial head. In this study, Mimics software was used to assign different material properties to different parts according to the CT gray values of the radial head. Based on the image data, the gray values (in Hounsfield units) were calculated for each cell in the mesh, and then the corresponding materials were defined according to different grayscale ranges. The software can accurately and efficiently distribute the model according to the gray values. Hence, this approach can accurately reflect the distribution of bone tissue in the proximal humerus, and simulating screw fixation at different angles with such a model can realistically reflect the stresses in the screw and surrounding bone tissue. The FE model can be used to analyze the stress and displacement distributions in the radial head and screws under different conditions and obtain accurate results. To the author's knowledge, no FE models have been developed to analyze the fixation of radial head fracture with absorbable screws.

### *Stress and Maximal Displacement*

**Fig. 6:**
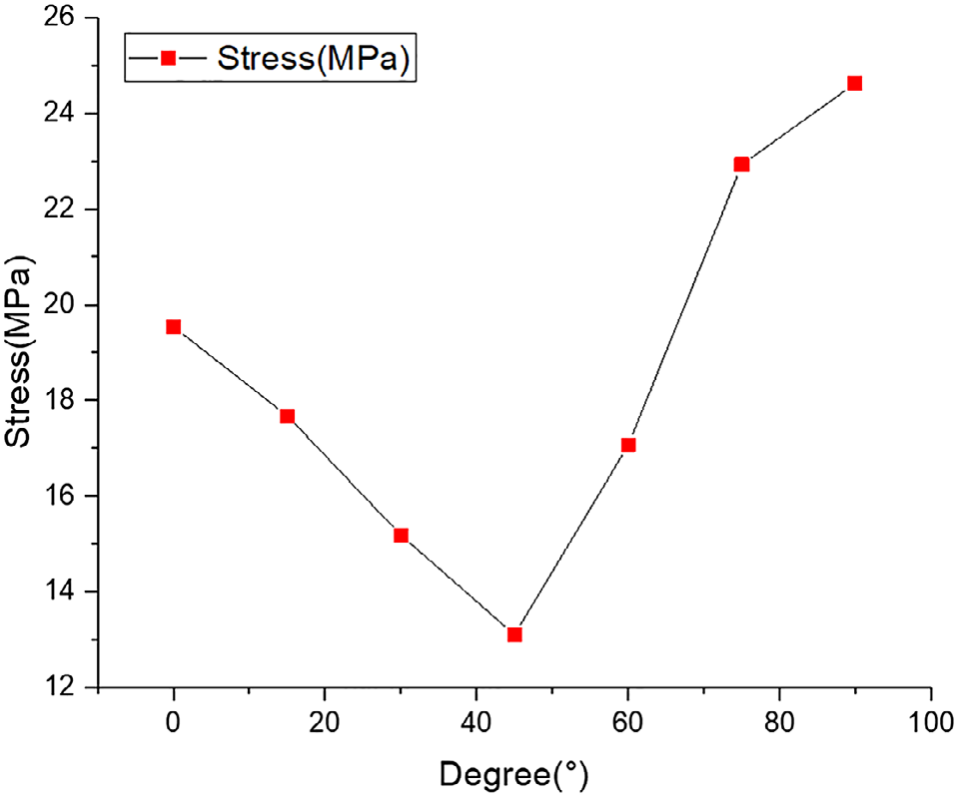
Comparison *of the Von Mises stress peaks of the screw under different insertion angles.*

**Fig. 7:**
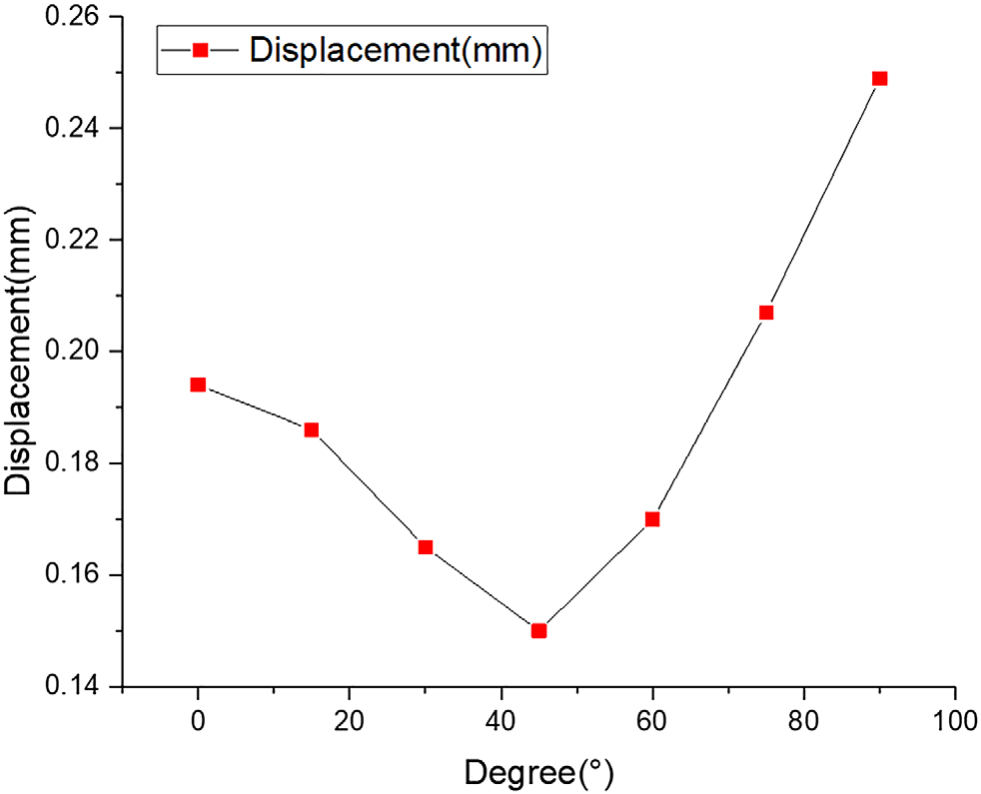
Comparison *of the peak displacements of the screw under different insertion angles.*

According to our results, the peak displacements and von Mises stress in the screws are presented in Figs 6 and 7, respectively. The von Mises stress in the screw and the fracture location were concentrated between the hole and the thread under the screw, whereas the maximum stress in the bone tissue around the screw was concentrated at the surface of the bone tissue. The stress between the screws decreased gradually when the angle increased from 0° to 45°, whereas the stress decreased when the angle increased from 45° to 90°. When the angle was 45°, the von Mises stress and peak displacements reached minimum values of 13.11 MPa and 0.15 mm, respectively. When the screw angle was 90°, the stress and peak displacements obviously increased, reaching up to 24.63 MPa and 0.25 mm, respectively; these conditions can destabilize the fracture block and increase the stress concentration. The reason for this phenomenon is that after increasing the angle between the screws, the screws will go through the radius, reducing the effective fixed length, increasing the stress, and increasing the displacement. The mechanical properties on the perforated side are reduced, which not only reduces the fixation effect but may even lead to bone rupture during drilling, causing secondary injury. When there is excessive screw length, the contralateral cortex may be penetrated, resulting in joint capsule damage or affecting joint rotation. When the angle between the two screws was 45°, the contact area around the screw thread was evenly and equally distributed. In contrast, the contact area around the screw thread was uneven when the angle was 90°, which may be related to the different stresses generated by the screws and the surrounding bone tissue when the screws were placed at different angles. The maximum screw displacement was not more than 0.25 mm, which had a very small effect on the patient, indicating that the fixation of radial head fractures with absorbable screws was reliable. When the angle was 45°, the stress was minimal, and subsequently the displacement was minimal, indicating that this angle provided the best stability. In this study, screws placed at 45° on the bone surface and the surrounding bone tissue showed a lower stress distribution than screws placed at other angles, which is similar to the results reported by Shi *et al*.^25^

Shi *et al*. used two screws (Wright, Beijing, China) to fix a fracture model, wherein they used three different screw orientations and pointed out that divergent screws showed the greatest axial stiffness in Mason type II radial head fractures. However, the article did not show exactly what angle between the screws can produce the optimal effect on stiffness. The research in this paper can solve this problem.

In the FEA, the maximum von Mises stress in the screws was 24.63 MPa when the angle was 90°, which was less than the shear and bending strength (176–215 MPa) of PLLA. The results show that absorbable screws can provide stronger stiffness to fix the fracture and minimize displacement, enabling earlier functional training to achieve postoperative rehabilitation. However, an FE model was established based on CT data of a type II radial head fracture, which could not represent the bone and tissue of other patients due to individual differences. Therefore, it is necessary to further improve this model and increase the sample size, which can be accomplished by combining cadaver experiments with the FE model to more closely approximate the real situation.

### *Limitations of the Study*

Although the previously mentioned findings of this study might be meaningful for clinical practice, some limitations of this study need to be mentioned. First, the standard bone without the joint capsule and other soft tissue attachment cannot simulate the force transmission and role of the real human elbow joint. Second, bone tissue and implants were defined as having linear‐elastic material properties. Because the focus of this research was not predicting the post‐yield mechanical behavior of the implants, isotropic linear‐elastic material models can be used to simulate the mechanical behavior prior to yielding. The tendency of the predicted results under various fixation conditions would not be substantially changed depending on the individual geometric model and simplified material properties. Moreover, the longitudinal load direction cannot completely simulate the biomechanics of all the daily activities of the elbow. Further clinical studies evaluating the findings of this study are also expected in the future.

## Conclusion

The primary stabilization of absorbable screw fixation for radial fractures was analyzed with an FE model. According to the numerically determined stress distribution, fixation with absorbable screws is safe for patients. When the angle between the two screws is 45°, the minimum von Mises stress can be generated, which should be recommended for type II radial fractures. Due to the resistance of screws to fracture, patients should be encouraged to ambulate early and perform rehabilitation activities after surgery. However, our conclusions need to be supported with a sufficiently large sample size and additional studies.

## References

[1] Rebgetz PR , Daniele L , Underhill ID , Ochsner A , Taylor FJ . A biomechanical study of headless compression screws versus a locking plate in radial head fracture fixation. J Shoulder Elbow Surg, 2019, 28: 111–116. 10.1016/j.jse.2018.10.008 30685273

[2] Gradl G , Jupiter JB . Current concepts review—fractures in the region of the elbow. Acta Chir Orthop Traumatol Cech, 2017, 9: 203–212. 22840951

[3] Kodde IF , Kaas L , Flipsen M , van den Bekerom MP , Eygendaal D . Current concepts in the management of radial head fractures. World J Orthop, 2015, 6: 954–960. 26716091 10.5312/wjo.v6.i11.954 PMC4686442

[4] Yoon A , Athwal GS , Faber KJ , King GJ . Radial head fractures. J Hand Surg Am, 2012, 37: 2626–2634. 23174078 10.1016/j.jhsa.2012.10.001

[5] Bryce CD , Armstrong AD . Anatomy and biomechanics of the elbow. Orthop Clin N Am, 2008, 39: 141–154. 10.1016/j.ocl.2007.12.001 18374805

[6] Swensen SJ , Tyagi V , Uquillas C , Shakked RJ , Yoon RS , Liporace FA . Maximizing outcomes in the treatment of radial head fractures. J Orthop Traumatol, 2019, 20: 15–23. 30904970 10.1186/s10195-019-0523-5 PMC6431334

[7] Demiroglu M , Ozturk K , Baydar M , *et al* Results of screw fixation in Mason type II radial head fractures. SpringerPlus, 2016, 5: 545–551. 27186508 10.1186/s40064-016-2189-2 PMC4848274

[8] Shikinami Y , Okuno M . Bioresorbable devices made of forged composites of hydroxyapatite (HA) particles and poly‐L‐lactide (PLLA): part I. basic characteristics. Biomaterials, 1999, 20: 859–877. 10226712 10.1016/s0142-9612(98)00241-5

[9] Fu XM , Oshima H , Araki Y , *et al* A comparative study of two types of sternal pins used for sternal closure: poly‐L‐lactide sternal pins versus uncalcined hydroxyapatite poly‐L‐lactide sternal pins. J Artif Org, 2013, 16: 458–463. 10.1007/s10047-013-0727-z 23996506

[10] Tarallo L , Mugnai R , Rocchi M , Capra F , Catani F . Comparison between absorbable pins and mini‐screw fixations for the treatment of radial head fractures Mason type II‐III. BMC Musculoskelet Disord, 2018, 19: 94–100. 29587695 10.1186/s12891-018-2014-x PMC5872384

[11] Wake H , Hashizume H , Nishida K , Inoue H , Nagayama N . Biomechanical analysis of the mechanism of elbow fracture‐dislocations by compression force. J Orthop Sci, 2004, 9: 44–50. 14767704 10.1007/s00776-003-0735-6

[12] Takatori K , Hashizume H , Wake H , Inoue H , Nagayama N . Analysis of stress distribution in the humeroradial joint. J Orthop Sci, 2002, 7: 650–657. 12486468 10.1007/s007760200116

[13] Strafun S , Levadnyi I , Makarov V , Awrejcewicz J . Comparative biomechanical analysis of stress‐strain state of the elbow joint after displaced radial head fractures. J Med Biol Eng, 2018, 38: 618–624. 30100829 10.1007/s40846-017-0334-1 PMC6061104

[14] Tan J , Mu M , Liao G , Zhao Y , Li J . Biomechanical analysis of the annular ligament in Monteggia fractures using finite element models. J Orthop Surg Res, 2015, 10: 30–40. 25890110 10.1186/s13018-015-0170-3 PMC4354748

[15] Langohr GD , Willing R , Medley JB , King GJ , Johnson JA . Contact analysis of the native radiocapitellar joint compared with axisymmetric and nonaxisymmetric radial head hemiarthroplasty. J Shoulder Elbow Surg, 2015, 24: 787–795. 25725964 10.1016/j.jse.2014.12.011

[16] Szmit J , King GJW , Johnson JA , Langohr GDG . The effect of stem fit on the radiocapitellar contact mechanics of a metallic axisymmetric radial head hemiarthroplasty: is loose fit better than rigidly fixed?. J Shoulder Elbow Surg, 2019, 28: 2394–2399. 31371158 10.1016/j.jse.2019.05.019

[17] Min W , Munro M , Sanders R . Stabilization of displaced articular fragments in calcaneal fractures using bioabsorbable pin fixation: a technique guide. J Orthop Trauma, 2010, 24: 770–774. 21076250 10.1097/BOT.0b013e3181ca38ce

[18] Caputo AE , Mazzocca AD , Santoro VM . The nonarticulating portion of the radial head: anatomic and clinical correlations for internal fixation. J Hand Surg Am, 1998, 23: 1082–1090. 9848563 10.1016/S0363-5023(98)80020-8

[19] Lipman MD , Gause TM , Teran VA , Chhabra AB , Deal DN . Radial head fracture fixation using tripod technique with headless compression screws. J Hand Surg, 2018, 43: 575.1–575.6. 10.1016/j.jhsa.2018.03.009 29709352

[20] Zwingmann J , Welzel M , Dovi‐Akue D , Schmal H , Sudkamp NP , Strohm PC . Clinical results after different operative treatment methods of radial head and neck fractures: a systematic review and meta‐analysis of clinical outcome. Injury, 2013, 44: 1540–1550. 23664241 10.1016/j.injury.2013.04.003

[21] Zhang J , Ebraheim N , Lause GE , Xiao B , Xu R . A comparison of absorbable screws and metallic plates in treating calcaneal fractures: a prospective randomized trial. J Trauma Acute Care Surg, 2012, 72: 106–110. 22439244 10.1097/ta.0b013e3182231811

[22] Zhang J , Xiao B , Wu Z . Surgical treatment of calcaneal fractures with bioabsorbable screws. Int Orthop, 2011, 35: 529–533. 21207026 10.1007/s00264-010-1183-5 PMC3066330

[23] Pelto K , Hirvensalo E , Bostman O , Rokkanen P . Treatment of radial head fractures with absorbable polyglycolide pins: a study on the security of the fixation in 38 cases. J Orthop Trauma, 1994, 8: 94–98. 8207581 10.1097/00005131-199404000-00003

[24] Wagner FC , Feucht MJ , Konstantinidis L , *et al* Biomechanical dynamic comparison of biodegradable pins and titanium screws for operative stabilization of displaced radial head fractures. Proc Inst Mech Eng H, 2020, 234: 74–80. 31702442 10.1177/0954411919884794

[25] Shi X , Pan T , Wu D , *et al* Effect of different orientations of screw fixation for radial head fractures: a biomechanical comparison. J Orthop Surg Res, 2017, 12: 143–147. 28969668 10.1186/s13018-017-0641-9 PMC5625609

